# Apolipoprotein Gene Polymorphisms (APOB, APOC111, APOE) in the Development of Coronary Heart Disease in Ethnic Groups of Kazakhstan

**DOI:** 10.4172/2157-7412.100021610.4172/2157-7412.1000216

**Published:** 2014-01-24

**Authors:** S Berkinbayev, M Rysuly, A Mussayev, K Blum, N Baitasova, A Mussagaliyeva, G Dzhunusbekova, B Makhatov, AA Mussayev, A Yeshmanova, R Lesbekova, Y Marchuk, R Azhibekova, M Oscar-Berman, M Kulmaganbetov

**Affiliations:** 1 Scientific Research Institute of Cardiology and Internal Diseases, Almaty, Kazakhstan; 2 Kazakh National Medical University named after S.D. Asfendiyarov, Almaty, Kazakhstan; 3 Department of Psychiatry and McKnight Brain Institute, University of Florida, College of Medicine, Gainesville, Fl., USA; 4 Dominion Diagnostics, LLC., North Kingstown, Rhode Island, USA; 5 Department of Physiology KazNAU, Almaty, Kazakhstan; 6 National Research Cardiac Surgery Center, Department of interventional cardiology, Astana, Kazakhstan; 7 Department of Psychiatry Anatomy and Neurology, Boston University School of Medicine and Veterans Administration System, Boston, Massachusetts, USA

**Keywords:** Molecular Genetics, Hypertriglyceridemia, Coronary Heart Disease (CHD; Apolipoprotein Gene Polymorphisms (APOB, APOC111, APOE), Kazakhs or Uyghurs

## Abstract

**Background:**

Previous Analysis of polymorphism of genes associated with the development of coronary heart disease (CHD) reveals that the frequency distribution of genotypes and alleles depends on the ethnic characteristics of the populations under study. Further impetus is derived from the well -established links between alcoholism (high prevalence in Kazakhstan region) and cardiovascular disorders.

**Objectives:**

The purpose of this study was to examine a number of apolipoprotein gene polymorphisms and correlate these alleles with changes of lipid profile in CHD patients of Kazakh and Uyghur nationalities.

**Methods:**

Four-Hundred Forty Eight (448) males of Kazakh and Uyghur nationalities residing in Kazakhstan were evaluated and genotyped. The age range of these subjects was 30–55 years which included both afflicted and controls. Specifically, 161- Kazakhs suffered from myocardial infarction compared to 112 health controls; 80- Uyghurs suffered from CHD compared to 95 health controls. Blood lipid profiles were examined in the total cohort. Genotyping was performed by polymerase chain reaction (PCR) using oligonucleotide primers identifying; ApoB; ApoC111; and APOE gene polymorphisms.

**Results:**

Initial screening revealed a significant inter-ethnic difference on the frequency of alleles associated with both the ApoB and APOE genes. We found that the X1 ApoB gene polymorphism is overrepresented in healthy Kazakhs relative to Uyghurs [86.4% in Kazakhs vs. 69.4% in Uyghurs]. Moreover, we found that the E4APOE allele was also overrepresented in healthy Kazakhs relative to Uyghurs [16.8% in Kazakhs vs. 9.5% in Uyghurs]. There was a significant relationship of polymorphisms of APOE such as ApoB and ApoC 111 with the value of lipid indices in Kazakhs. Additionally, we found that the E4 allele of the APOE gene also correlated with the value of lipid indices in Kazakhs. Further evaluation showed that the X2 allele of the ApoB and the S2 allele of the ApoCIII gene significantly associated with the lipid indices of Uyghurs.

**Conclusion:**

This systematic investigation confirms the association of various alleles of Apolipoprotein gene polymorphisms and contribution to aberrant lipid metabolism. Putatively at least in our population we are proposing that certain gene polymorphisms of Apolipoprotein genes such as ApoB; ApoC111; APOE ; X2 of ApoB; and S2 of ApoCIII differentially represented in either Kazakhs or Uyghurs are genetic markers of hypertriglyceridemia.

## Introduction

### Urgency of an issue

There is ample evidence linking high alcohol intake and cardiovascular disease throughout the world. Importantly, the prevalence rate of alcoholism in the Kazakhstan region and subsequent suicide is rather high and provides a rationale to evaluate genetic antecedents for coronary heart disease (CHD) [1]. It is known that the development of coronary heart disease depends in part on the differential expression of ethnic related gene polymorphisms [2]. Thus in terms of evolutionary genetics certain gene pools across various global populations due to inbreeding provide uniqueness with potential impact for a number of disease sates including CHD. A recent PUBMED search (12-2-13) revealed a remarkable list of 15,564 papers related to the search term “genes and lipid metabolism.” Interestingly, several biologic systems contribute to the pathophysiology of atherosclerosis and its complications, and within each of these systems many polymorphic genes have been linked to their variability in CHD risk [3]. Primarily they include genes of apolipoproteins, i.e. proteins involved in the formation of the lipoprotein particles of different density [4]. Taking into consideration the fact that dyslipidemia as a leading risk factor for development of CHD displays a genetic predisposition; the study of allelic variants of genes that are potentially most responsible for the phenotypic traits signifies the urgency of the issue.

### Hypothesis and objective

Based on many reviews and controversy related to the importance of genetic testing to determine predisposition of CHD risk [5], we hypothesized those two populations, specifically, Kazakhs and Uighurs may have differential polymorphisms of various apolipoproteins genes. We further hypothesized that these specific gene polymorphisms and resultant alleles may differentially load onto changes in the lipid spectrum, having an effect on CHD risk. Thus the objective of this work is to analyze through genotyping the relationship of polymorphisms of apolipoprotein genes with changes in the lipid spectrum in both Kazakhs and Uighurs in healthy controls and CHD as well myocardial infarction patients.

## Materials and Research Methods

### Subjects

Four-Hundred Forty Eight (448) males of Kazakh and Uyghur nationalities residing in Kazakhstan were evaluated and genotyped. The age range of these subjects was 30–55 years which included both afflicted and controls. These participants were all living in Kazakhstan, and unrelated by ties of kinship. Each subjects was selected by a by random sampling technique and subsequently interviewed. The University ethics committee approved this study as well as approving a consent form which was signed by each subject selected for the study.

### Diagnostic Characteristics

Specifically, 161- Kazakhs suffered from myocardial infarction compared to 112 health controls; 80-Uyghurs suffered from CHD compared to 95 health controls. The average age of the Kazakhs with CHD was 46.4 ± 1.9 years, of the apparently healthy Kazakhs – 40.7 ± 2.1 years, of the Uighurs with CHD – 47.3 ± 0.92 years, of the apparently healthy Uighurs – 41.2 ± 1.02 years.

### Laboratory blood tests

Standard blood lipid profiles were examined in the total cohort. The blood lipid profiles analyzed is as follows: total cholesterol (TC) and high-density lipoprotein cholesterol (HDLC), low density lipoprotein cholesterol (LDLC), triglycerides (TG). It is noteworthy, that analysis of all blood lipid profiles, were determined by a biochemical analyzer utilizing well-known procedures [6]. In addition, Apolipoproteins (Apo B, Apo C111, Apo E) were determined by immune-electrophoresis.

### Genotyping

Genotyping was performed by polymerase chain reaction (PCR) using oligonucleotide primers identifying; ApoB; ApoC111; and APOE gene polymorphisms. DNA was isolated from blood lymphocytes by a standard technique [7] to investigate polymorphisms of the Apo B, Apo CIII, Apo E genes. All DNA fragments including analyzed polymorphisms were amplified in the automatic thermal cycler “Gene Cycler” (Bio-Rad, USA) using the thermo stable Taq polymerase [8].Primers were used during amplification to obtain the necessary DNA fragments for subsequent analysis. We have performed the mathematical Hardy–Weinberg principle to determine that allele and genotype frequencies in a population remained constant from generation to generation in the absence of other evolutionary influences. We found satisfaction that we adhere to the Hardy– Weinberg equilibrium, model.

### Allelic description

For the genotyping of the Apo C111, the products of polymerase chain reaction (PCR) were digested with SStI, and then analyzed in 10% polyacrylamide gel. Alleles not having the SStI restriction site, were designated as S1, and the site carrying SStI – as S2. The combination of these alleles, form three genotypes: S1S1; S1S2; S2S2.

For the genotyping of the Apo B, the resultant material derived from amplification was digested with Xba I. The restriction products were analyzed utilizing electrophoresis in 12% polyacrylamide gel. As a result, two alleles were identified: XI – without a restriction site, X2 – with the restriction site for Xba I.

For the genotyping of the Apo E, the amplification product was digested with Hhal utilizing an improved method [9] and analyzed in 12% polyacrylamide gel. The method of genotyping of the Apo E identifies six common genotypes: E3/3, E2/2, E4/4, E2/3, E2/4, and E3/4.

## Results

### Plasma measurements

Following plasma measurements (mg/dl) we found that dyslipidemia atherogenicity (DA) is due not only to the buildup of lipid content, but is a function of the apolipoprotein components.

We observed a decrease of level of the Apo A1 and increase of level of the Apo B in the Kazakhs as well as Uighurs with CHD, and the indices of the Apo A1 are more prevalent and the indices of the Apo B of blood serum are lower in healthy individuals. While the indices noted for the Kazakhs were found to be significant for Apo A1 (<0.001) and Apo B (<0.05) for the Uighurs we found less significance for Apo A1 (<0.05) than in the Kazakhs but the significance for Apo B (<0.05) was the same for both populations (Tables 1 and 2).

As displayed in tables 1 and 2, the Apo E in the Kazakhs with CHD is significantly (<0.05) lower than in healthy individuals. There were not any differences in the levels of the Apo E in the compared groups of the Uighurs. There were ethnic differences in content of apolipoproteins: levels of the Apo A1, Apo B, Apo E were significantly (<0.05) higher among the Kazakhs over the Uighurs.

There is a plethora of population genetics throughout many countries regarding apolipoprotein genetic polymorphisms and structural relationships [10–14].

### Apo B gene

Following genotyping and analysis of the allelic frequency distribution of the Apo B gene we have found that the X1 allele of the Apo B gene is the most common allele in the Kazakhs and Uighurs (Table 3). However, there was no significant difference between CHD patients in Kazakhs and Uighurs.

### Apo B genotypes and plasmalipids

Since there is a large amount of research devoted to the study of associations of the Xba I polymorphism of the Apo B gene with atherogenic changes in lipid spectrum [15,16], we decided to analyze the lipid and apolipoprotein blood spectrum with different genotypes of the Apo B gene in patients with CHD and healthy individuals living in Kazakhstan.

Our analysis (Table 4) revealed that there were no significant differences observed for any measured plasma lipid level (A1, B and E apolipoproteins) relative to any ApoB allele (X1X1, X1X2, X2X2) suggesting that the Xba1 ApoB polymorphism did not affect the function of the lipid transport system.

On the contrary, in the Uighurs with CHD our analysis (depending on the availability of the restriction site) showed (Table 5) that in the group of homozygotes on the X2 allele (X2X2) of the Apo B gene the level of total cholesterol (TC), the low density lipoprotein cholesterol (LDLC) of the atherogenic index (AI) and the B apolipoprotein was significantly higher (p < 0.05) than in the heterozygotes X1X2 and homozygotes X1X1. Thus from this analysis the X2 allele of the Apo B gene is positively associated with the atherogenic shift.

### Apo C111 genotypes andplasmalipids

The Apo C111 gene produces a protein product that plays an important role in the metabolism of lipoprotein atherogenic fractions. Seven polymorphisms of the Apo CIII gene are described [17]. The most widely studied in this regard is the Sst 1 polymorphism in the 3' untranslated region of the Apo CIII gene caused by replacement of cytosine to guanine, resulting in the formation of a site for the action of Sst I restriction endonuclease [18]. Polymorphism on the Sst 1 site of the Apo C111 gene identifies two alleles: S1 – absence of a restriction site and S2 – presence of a restriction site. Analyzing these data it can be noted that the S1 and S2 allele frequencies of the Apo C111 gene vary considerably in different races.

Based on our genotyping data most of the surveyed individuals of Kazakh and Uighur ethnic groups are the carriers of the S1 allele of the Apo CIII gene. Among healthy Kazakhs the frequency of this allele is 76.6%, and among healthy Uighurs is 74.5% (Table 6).

When comparing the frequency distribution of the alleles of the Apo CIII gene between the groups of patients with CHD and healthy individuals we have not found any differences among the Kazakhs and the Uighurs.

To determine the effect of the Apo CIII gene polymorphism on the levels of the lipid and apolipoprotein blood spectrum in the Kazakhs and Uighurs, we analyzed the average values of these parameters with different genotypes (S1S1, S1S2, S2S2) of the Apo CIII gene (Tables 7 and 8). When comparing the lipid parameters of patients with CHD in terms of the Apo CIII gene polymorphism, we found that in the Kazakhs with the S1S1 genotype the triglyceride level was significantly increased in comparison with the S1S2 heterozygotes (2,5 ± 0,07 mmol/l) and homozygotes on the S2 alleles (S2S2 2,1 ± 0,08 mmol/l), and was (2,8 ± 0,10 mmol/l, p < 0.05).

We also found for VLDLC (Very Low Density Lipoprotein Cholesterol): its highest value was determined by the SISI genotype, the smallest – by the S2S2 genotype, the average – by the S1S2 genotype. Significant differences were not found in the Kazakhs compared by genotype with respect to levels of the TC, LDLC, HDLC, AI and levels of the A1, B and E apolipoproteins.

In contrast, the increased level of triglycerides was associated with subjects processing the S2 allele in the Uighurs with CHD (Table 8). Significant difference (P<.05) with the S1S1, SIS2, S2S2 genotypes was found in terms of the level of VLDLC: it was the highest in the examined subjects with the S2S2 genotype.

However, as depicted in Table 8 we did find that the Sst I polymorphism of the Apo CIII gene (i.e. carrier of the S2allele) is associated with the increased level of triglycerides and with the frequency of CHD in Uighurs.

### Apo E gene

Effect of genetic polymorphism of the Apo E gene on the risk of development of CHD has been extensively studied. However, in terms of our population the prevalence of this gene and associated polymorphisms remains unknown.

Thus in our study carriers of the E2/E2 genotype were not found among the Kazakhs and the E4/E4 and E2/E4 genotypes were not found in the group of healthy individuals. However, the E3/E3 genotype was the most common genotype in the Uighurs, the E4/E4 genotype was not found, and the E2/E2 genotype was found in only one patient and as such is a rare variant. Thus a high frequency of the E3 allele was observed in the examined groups of Kazakhs and Uighurs, and the frequency of the E4 allele is greater than or close to the frequency of the E2 allele.

It is known that among many of the common polymorphisms the Apo E is an allele polymorphism shown to consistently affect lipids in all populations studied, especially on total cholesterol and triglycerides. The E4 allele is more often found in patients with hypercholesterolemia, whereas the E2 allele – in patients with hypertriglyceridemia. Taking into consideration these data, we conducted a study of the relationship of polymorphisms of this gene with the lipid and apolipoprotein spectrum.

We thus observed in Kazakhs with CHD that the increase of TC, LDLC, AI is a function of the E2/E3, E3/E3, E2/E4, E3/E4, E4/E4 genotypes (Table 9).

The average level of total cholesterol (5,9 ± 0,09 mmol/l and 5,7 ± 0,06 mmol/l) in the Kazakhs having the E4/E4 and E3/E4 genotypes is significantly increased in comparison with this parameter in patients with CHD having the E3/E3 and E2/E3 genotypes (5,0 ± 0,05 mmol/l and 4,82 ± 0,07 mmol/l) (p < 0.05). The lowest concentrations of LDLC are observed in patients having the E2/E3 genotype (3,45 ± 0,08 mmol/l) and the highest concentrations – in patients having the E4/E4 genotype (4,54 ± 0,14 mmol/l), [p < 0.05].

Finally, in the Uighurs with CHD the E2/E3 genotype is significantly associated only with hypertriglyceridemia (2,5 ± 0,04 mmol/l). In the case of other genotypes (E3/3, E3/4) of the Apo E gene such an association has not been found (Table 10).

## Discussion

The present work is based on the search of genetic markers of coronary atherosclerosis with the help of analysis of associations of polymorphisms of DNA – with three candidate genes of atherosclerosis.

### Apo B gene

One of the most common polymorphisms of the Apo B gene is the Xba1 restriction endonuclease polymorphism. The appearance of the site for this endonuclease gene in the 26-th exon of the gene is due to nucleotide substitution (T-C) in the third codon position 2488. Due to the results obtained we can conclude that the E2 allele of the Apo E gene is the risk factor for development of hypertriglyceridemia in the Uighurs with CHD. Moreover, the presence of the E4 allele of the Apo E gene is a genetic marker of predisposition to hypercholesterolemia in the Kazakhs with CHD.

As described in the literature, the XI allele frequencies are very close in the Caucasian populations and ranged from a minimum - 43.0 % in the Danes to the maximum - 56.0% in the Italians. The XI allele frequency in the Indians is much higher - 75.0 %. This parameter reaches a maximum in the Mongoloids - 90.0 %: in the Chinese - 92.0 %, in the Japanese - 95.0 %, to the total mono-morphism in the Koreans - 100 % [3–5].

Furthermore, the X1 allele frequency in the Kazakhs is close to the X1 allele frequency in other Mongoloids, while the X1 allele frequency in the Uighurs is close to this parameter in the population of the Hindus (75.0 %). The Uighurs polymorphisms are found in between the Hindus and Kazakhs as for the X1 allele frequency. Probably, such a variety of the distribution of alleles of the Apo B gene reflects not only the region of residence, but to a large extent ethnicity as well.

This genetic marker studied globally, has a different degree of association with cardiovascular diseases – based on presence or absence of the X1 and X2 alleles. Specifically, association of the Xba I polymorphism with the risk of CHD [17] was found in a number of European populations. In some other studies conducted in Europe, Russia, Japan, Korea, China the Xba I polymorphism was not associated with a predisposition to CHD [10–14].

Further confirmation of our present results will establish the fact that the Apo B gene polymorphism is not associated with the development of CHD among the Kazakhs and Uighurs. However, in the Uighurs with CHD the X2 allele of the Apo B gene marks the atherogenic shift of the lipid and apoprotein spectrum, which indicates a significant contribution of the Apo B gene in the determination of abrarrant lipid metabolism.

### Apo CIII gene

In the literature there are reports on the relationship of allelic frequency of the Apo CIII gene not only with the levels of parameters of blood lipid spectrum, but also with coronary atherosclerosis [19–22].For example, a high frequency of the SI allele of the Apo CIII gene was found in patients with hypertriglyceridemia living in Japan in comparison with the frequency of the S1 allele in normal lipid individuals [23–24].A number of investigations [25,26] revealed that the frequency of the S2 allele of the Apo CIII gene in patients with CHD of Caucasoid origin with angiographically documented atherosclerosis was significantly increased in comparison with the frequency of the S2 allele of a control group. Paulweber et al, [17] concluded that the Apo CIII gene polymorphism and the S2 allele may serve as a predisposition marker to coronary atherosclerosis. However, there is a paucity of data regarding frequencies of polymorphic alleles of the Apo CIII gene with CHD [27,28] in Scotland and Austria.

It is known that the distribution of the S1 and S2 alleles among the Caucasoid ethnic groups is varied. Frequency of the S1 allele varies from 87.0 in the Russians to 94.0% in the British, and frequency of the S2 allele – from 13.0 to 6.0% respectively [25,27,29]. The S1 allele frequency in the Japanese is 65.0 %, while in the Hindus - 73.0 %, and in the Koreans are intermediate - 70.0 % [23,24,30,31].

Since we have found no differences between any of the analyzed alleles of the Apo CIII gene in the Kazakhs and Uighurs, we are proposing that polymorphisms of this particular gene, is not associated with the development of CHD in this population. Our results are consistent with the results of studies conducted in the world. It is established that the S1 allele identified in the Apo C111 gene can be regarded as a risk genetic marker of predisposition to hypertriglyceridemia which is often linked to both atherosclerosis and CHD [32].

Our finding of an association of the Sst I polymorphism of the Apo CIII gene (i.e. carrier of the S2allele) with an increased level of triglycerides with the frequency of CHD in Uighurs is consistent with the literature. In fact similar associations have been found in other populations such as England, Canada and Finland, whereas Scottish descendants and some other ethnic groups showed negative associations [20,33,41].

Thus it is shown that the certain restriction polymorphisms of the Apo CIII gene in patients with CHD living in Kazakhstan are not associated with CHD. However, in particular based on these results, we are proposing that the S1 and S2 alleles of the Apo CIII gene may be considered as genetic markers of predisposition to hypertriglyceridemia in both the Kazakhs and Uighurs with CHD.

### Apo E gene

The largest study of MRFIT demonstrated the association of the Apo E4 allele not only with the increased risk of coronary atherosclerosis, but also with mortality [34,42].The frequency of alleles (E2, E3, E4) of the Apo E gene in individuals of different ethnic origins is not the same [35,36]. The frequency distribution of the Apo E alleles in Japan is as follows: 4% - E2, 85% - E3, 11% - E4 [37]. The Apo E4 allele with high frequency was found in the population of Finland. Accordingly, this could be one of the reasons for the high incidence of CHD in the Finnish in comparison with other populations of European countries [43]. The Apo E4 allele correlated with CHD in Japanese, Korean, Italian and British populations [39–41].

In general, the E4 allele is associated with high level of the TC, LDLC and the Apo B. The E4 allele correlates more likely than other isoforms with vascular lesions in early atherosclerosis, and is considered to be atherogenic. In contrast, the E2 allele in different populations is associated with the decrease of the Apo B, LDLC, and is considered to be anti-atherogenic [44].

Taking into consideration the universality of the Apo E protein, we must mention the role of the Apo E polymorphism in the development of Alzheimer’s disease. From 34 to 65% of these patients are carriers of the Apo E4 allele, while for the general population it is 24–31%. In all likelihood, this is due to the fact that the Apo E is the major apolipoprotein of human cerebrospinal fluid [43,45].The E3 allele is the most common; it is almost equally often found in both Europe and Asia [44,46].

Thus in our study, the Apo E gene polymorphism affects the development of CHD among the Kazakhs through interaction with the lipid metabolism system. It is appears that only the E4 allele is a reliable risk factor for development of hypercholesterolemia, increase of LDLC and AI in Kazakhs with CHD. Additionally, due to the results obtained we can conclude that the E2 allele of the Apo E gene is the risk factor for development of hypertriglyceridemia in the Uighurs with CHD.

Thus the presence of the E4 allele of the Apo E gene is a genetic marker of predisposition to hypercholesterolemia in the Kazakhs with CHD. However, the presence of the E2 allele is a genetic marker of predisposition to hypertriglyceridemia in the Uighurs with CHD.

## Conclusions

The polymorphisms of the Apo B, Apo C111, Apo E genes in the Kazakhs and Uighurs are informative for molecular genetic population and clinical studies. A significant inter-ethnic difference of the frequency distribution of alleles and genotypes of the Apo B, Apo E genes studied in ethnic groups has been found. The X1 allele of the Apo B gene and the E4 allele of the Apo E gene are determined more often in the Kazakhs than in the Uighurs.

There are significant association of alleles of the Apo E and Apo C111 genes with the value of lipid parameters in the Kazakhs, which confirms their substantial contribution to the determination of the abarrant lipid metabolism; the E4 allele of the Apo E gene is a leading molecular genetic factor in the development of hypercholesterolemia; the S1 allele of the Apo C111gene predisposes to the development of hypertriglyceridemia.

Significant association of alleles of the Apo B, Apo C111 and Apo E genes with the value of lipid parameters have been evaluated in the Uighurs, whereby we confirm the contribution of these genes as risk factors in the development of abarrant lipid metabolism. Carriage of the X2 allele of the Apo B gene significantly predisposes an individual to hypercholesterolemia and increased levels of LDLP and the Apo B.The S2 allele of the Apo C111 gene and the E2 allele of the Apo E gene are also genetic markers of hypertriglyceridemia.

Understanding these genetic antecedents in this population as risk factors for CHD may provide the physician with important and relevant facts to persuade both Kazakhs and Uighurs hard drinkers to significantly reduce their alcohol intake [1]. These data must be met with caution until they are further confirmed by other even larger studies as accomplished in other populations [47]. While we did obtain significance in a number of the association studies we cannot ascribe genetic power for these inferences from the current study. However, we are poised in further larger scale genetic experiments to provide clinically useful information in the future.

## Figures and Tables

**Table 1:** Apolipoproteins in the Kazakhs

Parameters	Patients with CHD		Healthy individuals		
(mg/dl)	(n=161)		(n=112)		P***
	M*	m**	M	M	
Apo AI	138.73	2.38	167.07	2.94	<0.001
Apo B	103.38	2.86	92.16	3.93	<0.05
Apo E	6.77	0.24	7.67	0.17	<0.05
Apo B/ Apo AI	0.76	0.02	0.56	0.03	<0.05

Notes:

* M - average indexes;

** m – deviations of indexes;

*** P – level of reliability

**Table 2:** Apolipoproteins in the Uighurs

Parameters	Patients with CHD		Healthy individuals		
(mg/dl)	(n=80)		(n=95)		P
	M	M	M	M	
Apo AI	128.66	2.96	139.13	2.9	<0.05
Apo B	89.66	2.91	74.93	2.94	<0.05
Apo E	3.98	0.11	4.06	0.1	>0.05
Apo B/ Apo AI	0.69	0.02	0.54	0.02	<0.05

Notes:

* M - average indexes;

** m – deviations of indexes;

*** P – level of reliability

**Table 3:** Genotypes and Alleles of the Apo B Gene in Patients with Coronary Heart Disease and Healthy Kazakhs and Uighurs

Genotypes and alleles	Kazakhs				Uighurs			
	Patients with CHD		Healthy individuals		Patients with CHD		Healthy individuals	
	n=161	Frequency, %	n=112	Frequency, %	n=80	Frequency, %	n=93	Frequency, %
Genotypes, %								
X1X1	108	67	83	74.3	48	59.7	44	47,2
X1X2	46	28.7	27	24.3	25	31.3	41	44,4
X2, X2	7	4.3	2	1.4	7	9	8	8,4
Alleles, %								
X1	262	81.4	193	86.4	121	75.4	129	69,4
X2	60	18.6	31	13.6	39	24.6	57	30,6

**Table:** 

Parameters (mmol/l)	X1X1 (n=108)	X1X2 (n=46)	X2X2 (n=7)	P1	P2	P3
TC (mmol/l)	5.30 ± 0.10	5.34 ± 0.13	5.39 ± 0.20	>0,05	>0,05	>0,05
HDLC	0.92 ± 0.03	0.90 ± 0.05	0.91 ± 0.08	>0,05	>0,05	>0,05
TG	2.23 ± 0.06	2.36 ± 0.08	2.40 ± 0.18	>0,05	>0,05	>0,05
LDLC	3.94 ± 0.08	3.97 ± 0.10	4.00 ± 0.20	>0,05	>0,05	>0,05
XCLPONP	0.44 ± 0.01	0.47 ± 0.02	0.48 ± 0.04	>0,05	>0,05	>0,05
AI (relative unit)	4.82 ± 0.19	4.93 ± 0.20	4.92 ± 0.31	>0,05	>0,05	>0,05
Apo A1 (mg/dl)	138.3 ± 2.4	139.4 ± 2.8	137.6 ± 4.1	>0,05	>0,05	>0,05
Apo B (mg/dl)	102.5 ± 2.9	104.8 ± 3.1	103.3 ± 4.2	>0,05	>0,05	>0,05
Apo E (mg/dl)	6.6 ± 0.2	6.9 ± 0.3	6.5 ± 0.4	>0,05	>0,05	>0,05

Note: P1 – Statistical differences of lipid parameters in patients with the X1X1 and X1X2 genotypes, P2 – Statistical differences of lipid parameters in patients with the X1X1 and X2X2 genotypes, P3 Statistical differences of lipid parameters in patients with the X1X2 and X2X2 genotypes.

**Table 5:** Relation of the Apo B Gene Genotype with the Lipid and Polipoprotein Spectrum of Blood in the Uighurs with Coronary Heart Disease (CHD)

Parameters (mmol/l)	X1X1 (n-48)	X1X2 (n-25)	X2X2 (n-7)	P1	P2	P3
TC (mmol/l)	5.50 ± 0.12	5.60 ± 0.15	6.2 ± 0.21	>0,05	<0,05	<0,05
HDLC	1.18 ± 0.04	1.19 ± 0.06	1.16 ± 0.11	>0,05	>0,05	>0,05
LDLC	3.91± 0.10	4.0 ± 0.13	4.64 ± 0.20	>0,05	<0,05	<0,05
VLDLPC	0.41 ± 0.02	0.41 ± 0.04	0.4 ± 0.05	>0,05	>0,05	>0,05
TG	2.04 ± 0.08	2.04 ± 0.09	1.98 ± 0.14	>0,05	>0,05	>0,05
AI	3.66 ± 0.14	3.71 ± 0.15	4.34 ± 0.23	>0,05	<0,05	<0,05
Apo A1 (mg/dl)	130.6 ± 2.2	128.1 ± 3.7	127.2 ± 4.1	>0,05	>0,05	>0,05
Apo B (mg/dl)	88.98 ± 2.63	93.09 ± 2.97	107.4 ± 3.45	>0,05	<0,05	<0,05
Apo E (mg/dl)	4.3 ± 0.10	3.9 ± 0.16	3.7 ± 0.28	>0,05	>0,05	>0,05

Note: P1 – Statistical differences of lipid parameters in patients with the X1X1 and X1X2 genotypes, P2 - Statistical differences of lipid parameters in patients with the X1X1 and X2X2 genotypes, P3 - Statistical differences of lipid parameters in patients with the X1X2 and X2X2 genotypes.

**Table 6:** Genotypes and Alleles of the Apo S111 in Patients with Coronary Heart Disease (CHD) and Healthy Kazakhs and Uighurs

Genotypes and alleles	Kazakhs				Uighurs			
	Patients with CHD		Healthy individuals		Patients with CHD		Healthy individuals	
	n=161	Frequency, %	n=112	Frequency, %	n=79	Frequency, %	n=95	Frequency, %
Genotypes, %								
S1S1	97	60	63	56.3	39	49.4	53	55.3
S1S2	50	31.1	45	40.6	35	44.3	36	38.3
S2,S2	14	8.9	4	3.1	5	6.3	6	6.4
Alleles, %								
S1	244	75.5	171	76.6	113	71.5	140	74.5
S2	78	24.5	53	23.4	45	28.5	48	25.5

**Table 7:** Relationship of the Apo C111 Genotype with the Lipid Spectrum in the Kazakhs with Coronary Heart Disease (CHD)

Parameters	SISI	SIS2	S2S2	P1	P2	P3
(mmol/l)	(n = 97)	(n = 50)	(n = 14)			
TC (mmol/l)	5.42±0.07	5.44±0.08	5.43±0.25	>0,05	>0,05	>0,05
HDLC	0.91±0.03	0.92±0.05	0.91±0.12	>0,05	>0,05	>0,05
TG	2.8±0.06	2.5±0.07	2.1±0.22	<0,05	<0,05	>0,05
LDLC	3.95±0.09	4.02±0.08	4.1±0.26	>0,05	>0,05	>0,05
VlDLPC	0.56±0.01	0.5±0.02	0.42±0.06	<0,05	<0,05	>0,05
AI (relative unit)	4.95±0.17	4.91±0.20	4.97±0.36	>0,05	>0,05	>0,05
Apo A1 (mg/dl)	136.74±2.63	138.0±2.88	139.8± 4.39	>0,05	>0,05	>0,05
Apo B (mg/dl)	105.1±2.87	104.0±2.96	103.5±4.36	>0,05	>0,05	>0,05
Apo E (mg/dl)	6.8±0.22	6.7±0.32	6.9±0.65	>0,05	>0,05	>0,05

Note: P1 – Statistical differences of lipid parameters in patients with the SISI and SIS2 genotypes, P2 – Statistical differences of lipid parameters in patients with the SISI and S1S2 genotypes, P3 – Statistical differences of lipid parameters in patients with the SIS2 and S2S2 genotypes.

**Table 8:** Relationship of the Apo C111 Genotype with the Lipid Spectrum in the Uighurs with Coronary Heart Disease (CHD)

Parameters	SISI	SIS2	S2S2	P1	P2	P3
(mmol/l)	(n =39)	(n = 35)	(n = 5)			
TC (mmol/l)	5.64±0.12	5.63±0.14	5.60±0.27	> 0,05	> 0,05	> 0,05
HDLC	1.18±0.03	1.17±0.04	1.15±0.09	> 0,05	> 0,05	> 0,05
TG	1.6±0.08	2.3±0.07	2.6±0.15	< 0,05	< 0,05	>0,05
LDLC	4.14±0.12	4.0±0.14	3.93±0.27	> 0,05	> 0,05	> 0,05
VLDLC	0.32±0.03	0.46±0.02	0.52±0.02	<0,05	<0,05	>0,05
AI (relative unit)	3.78±0.16	3.81±0.18	3.87±0.33	> 0,05	> 0,05	> 0,05
Apo A1 (mg/dl)	128.2±2.79	129.1±2.90	130.0±4.56	> 0,05	> 0,05	> 0,05
Apo B (mg/dl)	89.79±2.73	90.29±2.94	88.27±4.82	> 0,05	> 0,05	> 0,05
Apo E (mg/dl)	4.0±0.12	3.98±0.15	3.87±0.54	> 0,05	> 0,05	> 0,05

Note: P1 – Statistical differences of lipid parameters in patients with the SISI and SIS2 genotypes, P2 – Statistical differences of lipid parameters in patients with the SISI and S1S2 genotypes, P3 – Statistical differences of lipid parameters in patients with the SIS2 and S2S2 genotypes.

**Table 9:** Genotypes of the Apo E Gene and Parameters of the Lipid and Apolipoprotein Spectrum in the Kazakhs with Coronary Heart Disease (CHD)

Parameters (mmol/l)	E2E3	E3E3	E2E4	E3E4	E4E4
TC (mmol/l)	4.82±0.07	5.0 ±0.05	5.4±0.09*	5.7±0.06*	5.9±0.09*
HDLC	0.93±0.04	0.92±0.02	0.90±0.07	0.91±0.03	0.90±0.06
TG	2.2±0.06	2.1±0.07	2.3±0.10	2.4±0.08	2.3±0.15
LDLC	3.45±0.08	3.66±0.05	4.04±0.19*	4.31±0.07*	4.54±0.14*
VLDLPC	0.44±0.03	0.42±0.01	0.46±0.11	0.48±0.03	0.46±0.10
AI (relative unit)	4.18±0.18	4.43±0.13	5.0±0.26*	5.26±0.15*	5.56±0.22*
Apo A1 (mg/dl)	139.5± 3.6	138.6±2.3	138.4±4.3	137.9±3.2	136.3±5.5
Apo B (mg/dl)	104.1±3.8	105.1±2.4	103.1±4.4	100.2±2.9	101.9±4.2
Apo E (mg/dl)	6.6+0.32	6.8±0.25	6.4±0.48	6.3±0.28	6.2±0.40

* p< 0,05

**Table 10:** Genotypes of the Apo E Gene and Parameters of the Lipid and Apolipoprotein Spectrum in the Uighurs with Coronary Heart Disease (CHD)

Parameters (mmol/l)	E2E3	E3E3	E3E4
TC	5.60 ±0.16	5.58±0.14	5.69±0.24
HDLC	1.17±0.03	1.18±0.04	1.14±0.07
TG	2.5±0.04*	1.74±0.09	1.70±0.18
LDLC	3.95±0.19	4.05±0.15	4.21±0.22
VLDLPC	0.5±0.09*	0.35±0.02	0.34±0.04
AI (relative unit)	3.79±0.24	3.73±0.22	3.9±0.36
Apo A1 (mg/dl)	132.8±6.59	132.2±3.91	122.0±3.99
Apo B (mg/dl)	86.54±4.31	92.65±3.68	87.41±5.03
Apo E (mg/dl)	4.00±0.25	4.03±0.13	4.02±0.24

* -p < 0,05
